# Thermal inkjet bioprinting drastically alters cell phenotype

**DOI:** 10.1088/1758-5090/acd3b3

**Published:** 2023-05-25

**Authors:** Patricia Ablanedo Morales, Brittany Rodriguez, Michael E Furth, Kayla B Molina, Andrew J Boland, Jonaton E Mohl, Thomas Boland

**Affiliations:** 1 Biomedical Engineering, The University of Texas at El Paso, El Paso, TX, United States of America; 2 Department of Leadership Engineering, The University of Texas at El Paso, El Paso, TX, United States of America; 3 Department of Chemistry and Biochemistry, The University of Texas at El Paso, El Paso, TX, United States of America; 4 Biochemistry, The University of Texas at Austin, Austin, TX, United States of America; 5 Department of Mathematical Sciences, The University of Texas at El Paso, El Paso, TX, United States of America

**Keywords:** thermal inkjet bioprinting, pluripotency, differentiation

## Abstract

Since the first description of inkjet bioprinting of cells in 2003, quantifying the input and measuring the output of the printers has been the hallmark of the field of bioprinting, as it is virtually impossible to characterize cells that are inside the printing orifices or extrusion needles. We will describe here some recent discoveries of cell behavior due to inkjet bioprinting. Primary and immortalized adult dermal fibroblasts were expanded for 2–3 passages upon receiving. The cells were harvested, resuspended in PBS, and bioprinted into a 96-well plate with pluriSTEM media. Cells were then transferred either into precoated 96-well plates or 20 *µ*l drops were pipetted for hanging drop culture. IPC differentiation protocols were applied and the induction was begun approximately 45 min after printing. When differentiating aggregates, the initiation happened 45 min after the aggregates were transferred into the 96 wells. Standard immunostaining and RNA sequencing (RNA-Seq) were used to analyze the cell phenotypes. Preliminary results indicate that all cells expressed the three pluripotency markers oct-4, nanog, and sox-2. After applying a cardiomyocyte differentiation protocol, the cells stained positively for troponin-3. The cells also elongated and became more cardiomyocyte-like in their morphology. We analyzed bulk RNA seq data and our preliminary results show upregulation of some genes that have been implicated as stem cell markers: EPCAM, LEFTY1, ZFP42, and TEX19. In addition, differential expression of genes associated with pluripotency-relevant pathways shows some pathways are off like the MAPK/p38, MAPK/JNK1-3 which is expected for a pluripotent state. We also have data supporting the activation of the hippo pathway with transcriptional co-activator with PDZ binding motif (TAZ) highly upregulated and yes-associated protein staining the cell body. In addition, GSK3B is off and TGFB1, LIF/PIK3, and AKT1 are on as expected for pluripotency. Examining the gene network of upregulated genes, one can clearly distinguish the pivotal role of FOS, FOXO1, and PIK3 all related to pluripotency. Bioprinted fibroblasts will at least temporarily adopt a more primitive or dedifferentiated state, reminiscent of pluripotency. While immunochemistry shows the classic transcription factors required for pluripotency, gene expression shows a more nuanced picture of the transformations that occur upon printing. Understanding these transformations, even if temporary will be crucial when trying to build tissues using bioprinting technologies.

## 1. Introduction

Since the inception of bioprinting [1] more than a decade ago, much is still unknown regarding the cell biology of printed cells. Solis *et al* [2] documented expression levels of angiogenic factors in thermal inkjet bioprinted (TIB) and putative pathways activated by the printing process. Campbell *et al* [3] demonstrated far-reaching mRNA changes in TIB MCF-7, a breast cancer cell line, including highly upregulated expressions of IL-6, ALDH1A3, ALDH3A1, and CD44, all genes whose expressions have been associated with breast cancer stem cells. This was intriguing and led us to hypothesize that normal adult primary cells may also undergo sweeping changes in gene expressions upon TIB and may express genes that are associated with stem cells. As these and other papers have shown, mechanical stimuli can be powerful drivers of intracellular activities, including regulation of epigenetic functions (see, for example, a review by Lv *et al*) [4], yet most of the research on inducing stem cells focuses on the introduction of DNA into the cell using episomal vectors [5], Sendai virus vectors [6] or mRNA transfection technology [7], or on chemical stimuli driven reprogramming via administration of drugs, transcription factors, and other chemical cocktails [8]. We, therefore, studied the expression of pluripotent cell markers in freshly TIB fibroblasts, and we report results from a preliminary transdifferentiation experiment to induce the cardiomyocyte lineage in primary adult fibroblasts shortly post-TIB.

## 2. Materials and methods

### 2.1. Cell handling

Adult Human dermal fibroblasts (ATCC, PCS-201-012, Lot# 70017605 and ATCC HS27) were expanded for 2–3 passages upon receiving in complete medium, Dulbecco’s modified Eagle’s medium (DMEM) supplemented by 10% fetal bovine serum (FBS) and 1% antibiotic/antimycotic solution in a 5% CO2 incubator at 37 C. Upon growing around 80%–90% of confluency, the cells were rinsed with PBS, detached with trypsin, harvested, and centrifuged at 2000 rpm for 5 min. The supernatant was removed, the cell pellet was resuspended in media, and the cells were counted. Then the cells were centrifuged again and resuspended in PBS to yield a 500 *µ*l solution of approximately 1.6 × 10^6^ cells ml^−1^. Approximately half of the cells were manually pipetted into wells. The remaining half were pipetted into emptied and autoclaved inkjet cartridges (HP 33) adapted for a modified HP thermal inkjet printer and were used for bioprinting purposes [9]. Several control experiments were conducted to account for heat and shear. Cells were forced through the nozzles of the cartridge by attaching a syringe and pressurizing until a stream of droplets was seen to emerge from the nozzles. This eliminated the heating of the nozzles. In a similar fashion, cells were forced through the nozzles while they were immersed in media, thus eliminating drop formation while maintaining shear. In other experiments, the PBS-cell solution was printed into a 96-well plate, with each well filled with approximately 40 *µ*l of pluriSTEM media (Sigma-Aldrich, SCM130). Cells were then transferred either into precoated 96-well plates (Thermo Fisher Scientific, #1256670) or 20 *µ*l drops were pipetted onto the back of the top lid of a sterile 100 mm × 15 mm polystyrene petri dish (Fisherbrand, #0875713) for hanging drop culture using a multichannel pipettor [10]. The 96-well plates were precoated with a stem cell qualified ECM gel (Sigma-Aldrich, CC131-5ML) that was diluted 80x from the original concentration with DMEM/F12 (Sigma-Aldrich, D6421) using frozen pipettes and plates according to the manufacturer’s instructions at least 24 h prior to the printing experiments and kept in a refrigerator at 4 C. Approximately 45 min before the experiments, the plates were warmed up slowly to room temperature, and the media was exchanged with PluriSTEM. The lids with the drops were inverted and placed on their dish bottom, which contained several ml of PBS, and incubated. After 48 h, each of the hanging drops were added to a new precoated well of the 96-well plate then either fixed or the differentiation protocols applied.

### 2.2. Cell differentiation protocol

The Bhattacharya *et al* [11] differentiation protocol was followed to the utmost detail. Differentiation media 1 and 2 were prepared, and the induction was begun (day 0) approximately 45 min after TIB for the cells that were transferred to the plate immediately after printing. When differentiating aggregates, the initiation happened 45 min after the aggregates were transferred into the 96 wells. Note that we did not use the ROCK inhibitor prior to the Bhattacharya protocol’s induction. With this exception, the protocol was followed until days 10–12, when the cells were fixed.

### 2.3. Cell fixing, staining, and imaging

Cells were fixed by adding 100 *µ*l of 4% paraformaldehyde solution to each well of the 96-well plate (clear thin flat bottom TC-treated Imaging-Falcon 353 219) that contained cells to be fixed. After 20 min under the biosafety hood, the solution was gently aspirated, and wells were also gently rinsed 2x with 100 *µ*l PBS. The cells were permeabilized using 100 *µ*l 0.1% triton-X in PBS per well for 10 min, then twice aspirating and rinsing with 100 *µ*l PBS for 5 min and leaving the cells in PBS. For blocking purposes, we aspirated the PBS and added 100 *µ*l of 1% BSA, 22.52 mg ml^−1^ glycine in PBST (PBS+ 0.1% Tween 20) for 30 min for each well. After aspiration, the desired wells were filled with 50 *µ*l of PBST, and 1 *µ*l of Alexa-488 conjugated primary antibody against Oct-4 was added (clone 10H11.2, Alexa Fluor^®^488, Sigma-Aldrich, FCMAB113A4) and left for 1 h. After rinsing with PBS, the wells were filled with 50 *µ*l of PBST and 1 *µ*l of either Alexa Fluor^®^ 647 conjugated anti-Sox-2 (MD Millipore Corporation, AB5603-AF647) or Alexa Fluor^®^ 647 conjugated anti-Nanog (Cell Signaling Technology, 5448S) antibody for 1 h. After a second rinse with PBS, DAPI (Thermo Scientific, #62248, diluted 1:1000 in PBS) was added to the wells for 30 min, and the wells were rinsed once again and kept under 100 *µ*l of PBS in a refrigerator at 4 C until imaged. The yes-associated protein (YAP) was visualized by using a YAP primary antibody followed by an antigoat secondary antibody labeled with Alexa Fluor^®^ 647. The cells were counterstained with an anti alpha-tubulin antibody that was conjugated with Alexa Fluor^®^ 488 followed by DAPI. For differentiation experiments, the fixed, permeabilized, and blocked cells were incubated with Alexa Fluor^®^ 647 conjugated Troponin I Type 3 antibodies (Novus Biologicals, NB100-73087AF647) for 1 h at room temperature, then rinsed and filled with DAPI solution as described above. Control cells were treated in the exact same manner.

The wells were imaged using a Zeiss confocal and a Nikon confocal system. High-resolution digital fluorescent images were obtained on a Zeiss LSM 700 confocal microscope equipped with an EC Plan-Neofluar 40X/1.30 oil differential interference contrast (DIC) objective and assisted with ZEN 2009 software (Zeiss, New York, NY). Single-plane images were sequentially scanned using a 1-Airy unit (AU) pinhole setting for each channel and acquired at a 1024- by 1024-pixel resolution. The ZEN 2009 software was also employed to obtain the colocalization of the red, green, and blue signals and to export the images as TIFF files. Fluorescent images were also captured using a Nikon D-ECLIPSE C1si confocal microscope equipped with a Nikon Plan Fluor 10x/0.30 objective and a VC Plan Apo 20x/0.75 DIC objective in the EZ-C1 software suite. A 150 *µ*m pinhole setting was used in conjunction with the frame lambda functionality to capture sequential images of each channel at a 2048 by 2048-pixel resolution. A power intensity of 1% was used for the 408 nm laser and 10% was used for both the 488 nm and 565 nm lasers. The NIS-Elements software was then used to visualize the colocalization of the red, blue, and green signals and to then export the images as JPEG files.

### 2.4. RNA seq analysis

Bulk RNA sequencing was done at 2 h, and 12 h, post-TIB with the intention of identifying genes that were upregulated or downregulated with respect to the pipetted cells. The cells were detached with a cell sweeper to avoid exposure to influencing solutions, centrifuged at 800 rpm for 5 min followed by RNA extraction using the PureLink RNA mini kit (Thermo Fisher) following the manufacturer’s protocol. RNA concentration was evaluated with a Nano-drop 2000 spectrometer (Thermo Fisher Scientific) and the RNA Integrity Number equivalent was assessed for each sample and ranged from one to ten. RNA seq data was analyzed for data summarization, normalization, and quality control using Trimmomatic, Bowtie2 and DESeq2 [12, 13]. Differentially expressed genes were selected by using threshold values of >2 and < −2- fold change and a *q*-value ⩽0.05. A second analysis using the Log2 transformation was also conducted, the same threshold values as before, >2 and < −2 -fold change and a q-value ⩽0.05, were considered statistically significant. The *q*-value is used in genome-wide expression data, this statistical method is used to filter the proportion of false positives from a collection of *p*-values <> 0.05. STRING was used to map pathways of upregulated and downregulated differentially expressed genes. Cytoscape was used to conduct a network analysis to identify protein-protein interactions.

## 3. Results

**Figure 1.**
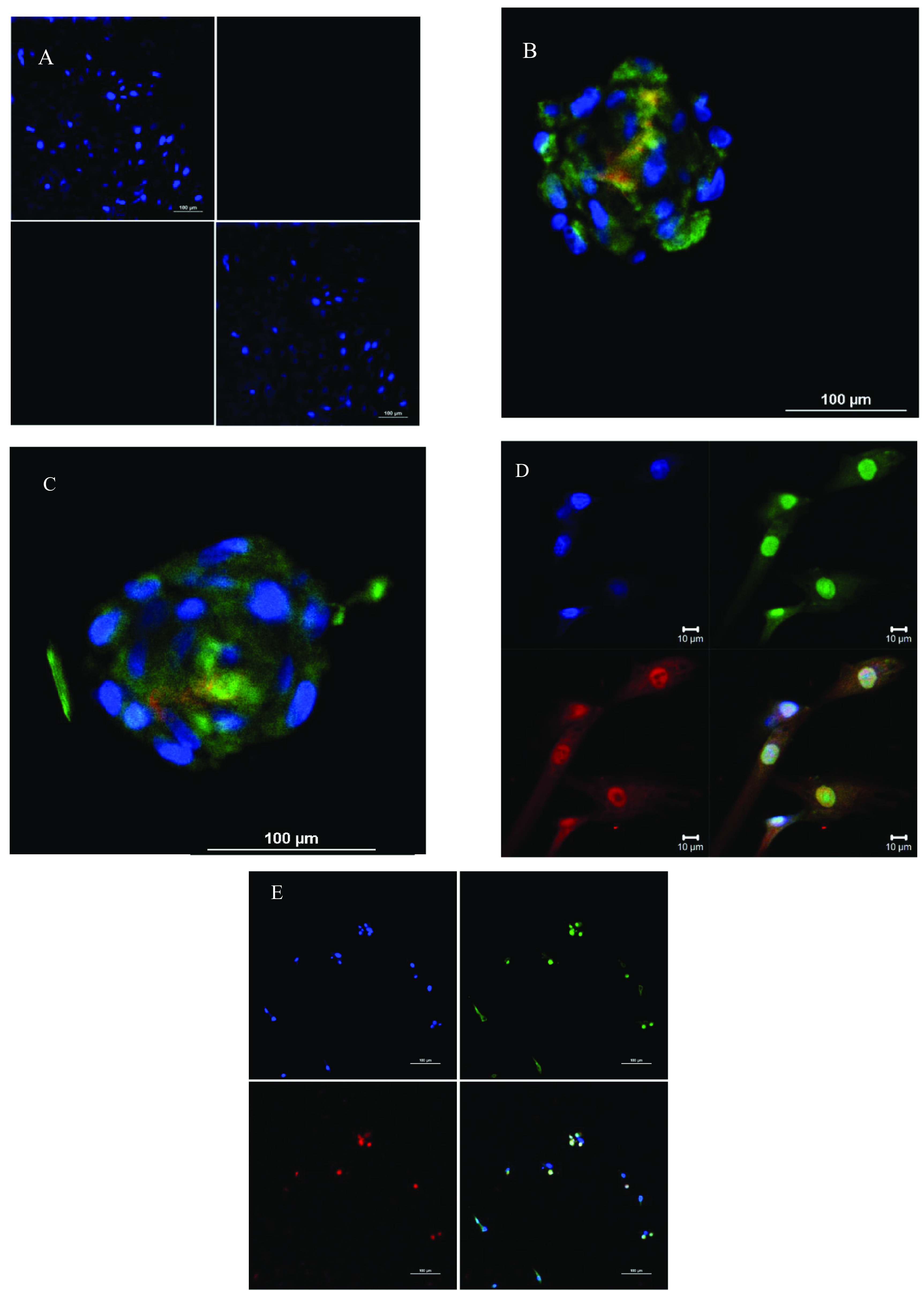
Confocal microscopy images visualizing staining for OCT-4, Sox-2 and Nanog proteins, as well as DAPI (A). Non-printed control samples (B). Cell aggregates after 50 h stained for OCT-4 (green), Sox-2 (red), and DAPI (blue) (C). Cell aggregates after 50 h stained for OCT-4 (green), Nanog (red) and DAPI (blue) (D). four-split panel of TIB cells plated after 24 h post-printing stained for OCT-4 (green), Sox-2 (red), and DAPI (blue) showing individual colors and all three color channels overlaid. E. four-split panel of TIB cells plated after 24 h post-printing stained for OCT-4 (green), Nanog (red), and DAPI (blue) showing individual colors and all three color channels overlaid. Scale bars are 100 *µ*m except for D.

Figure 1 shows antibody staining of TIB fibroblasts 24 h post-printing, labeled with fluorescently tagged primary monoclonal antibodies against OCT-4 (green), Sox2 (red), and Nanog (red). Control cells, which were not printed but otherwise received identical treatment, are also shown in the figure and do not show any staining. Also shown is the staining of cell aggregates, formed by the hanging drop method, and stained with the three antibodies. Similar as seen in the images of plated cells, OCT-4 is expressed throughout all cells, but the transcription factor Nanog seems not to be expressed in all of the cells and is concentrated on the inner mass of the aggregates. While Sox-2 is present in all plated cells, although it appears less expressed in the 2-day-old aggregates than in the freshly plated cells.

**Figure 2.**
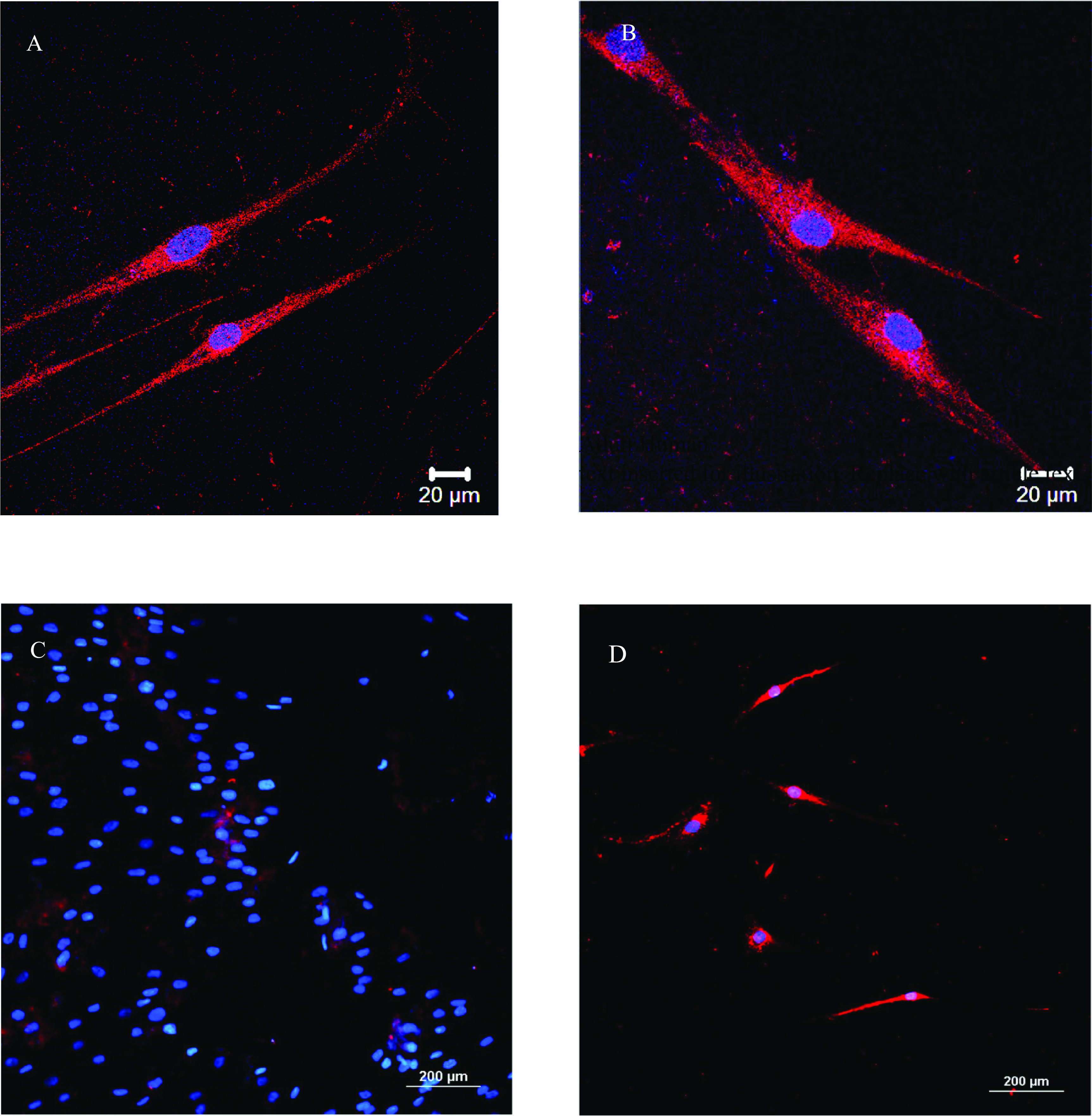
TIB fibroblasts were differentiated using a cardiomyocyte differentiation protocol. Cells were stained with a primary troponin I type 3 (cardiac) antibody labeled with Alexa Fluor 647. (A) and (B) a combined channel with cells that spread out from aggregates, as shown in figure 1 (B) and (C) generated by the hanging drop method. (C) Combined blue/red channel showing control samples showing non-printed cells that underwent the differentiation protocol. (D) Combined blue/red channel showing cells that were differentiated immediately after plating into a well without hanging drops.

Figure 2 shows TIB fibroblasts that were immediately after printing subjected to a cardiomyocyte differentiation protocol [11] Cells were stained with a primary troponin I type 3 (cardiac) antibody labeled with Alexa Fluor 647. Control samples with plated fibroblasts underwent the identical differentiation and staining protocols, but were not bioprinted are also shown and do not show any staining. A majority of the TIB cells are showing elongated, rectangular cell morphology and are positive for troponin I, much longer than the control cells. Cells seem to have elongated along a common axis and are seen interacting or connecting to each other in some of the images.

**Figure 3.**
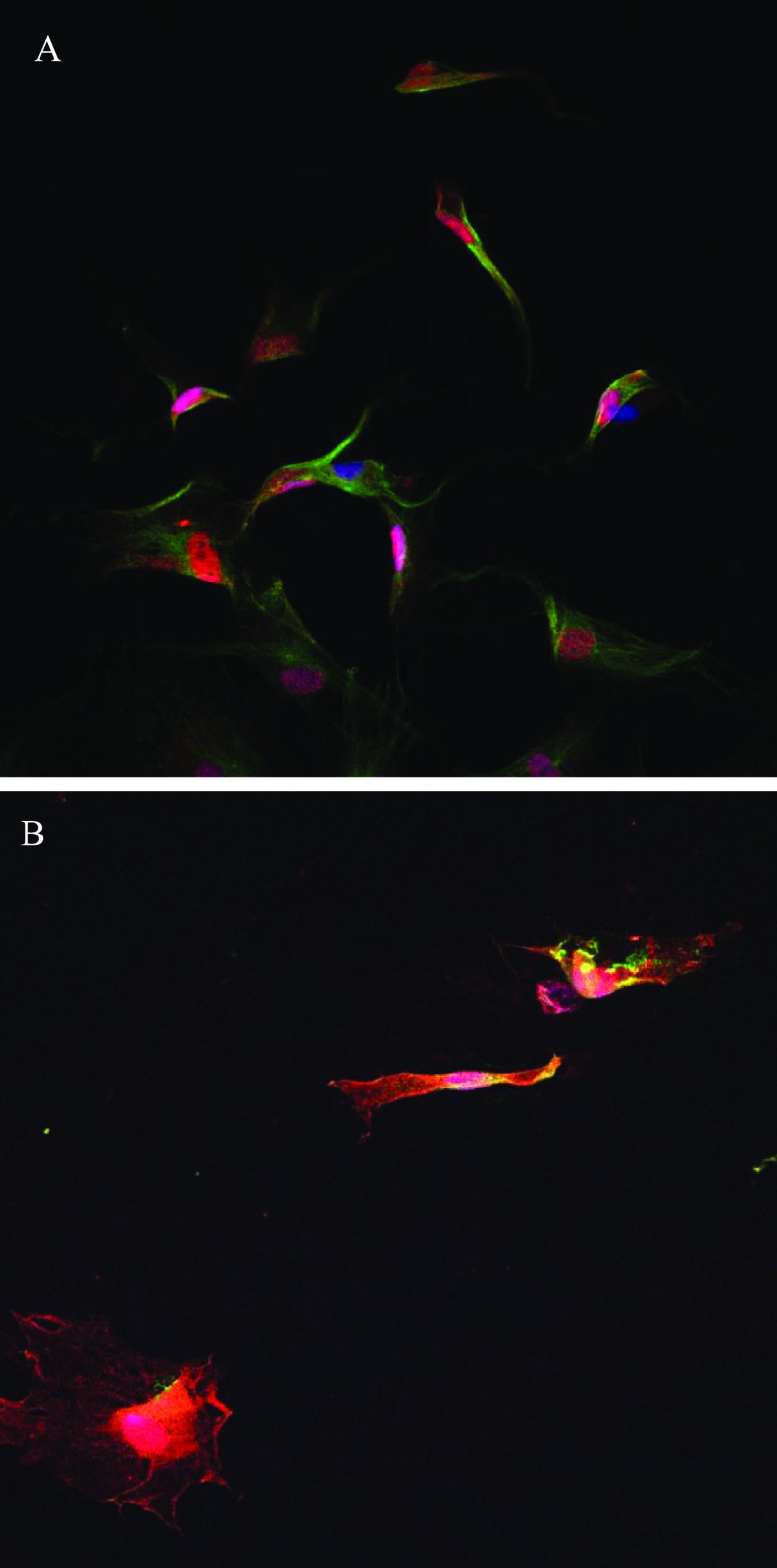
Shows TIB fibroblasts expressing YAP (red) and Alpha tubulin (green) after 3 h (A) and 24 h (B) postprinting.

Figure 3(A) shows TIB cells 3 h post-printing staining for YAP (red) alpha-tubulin (green) and nucleus (blue) while figure 3(B) shows the cells at 24 h post-printing.

**Figure 4.**
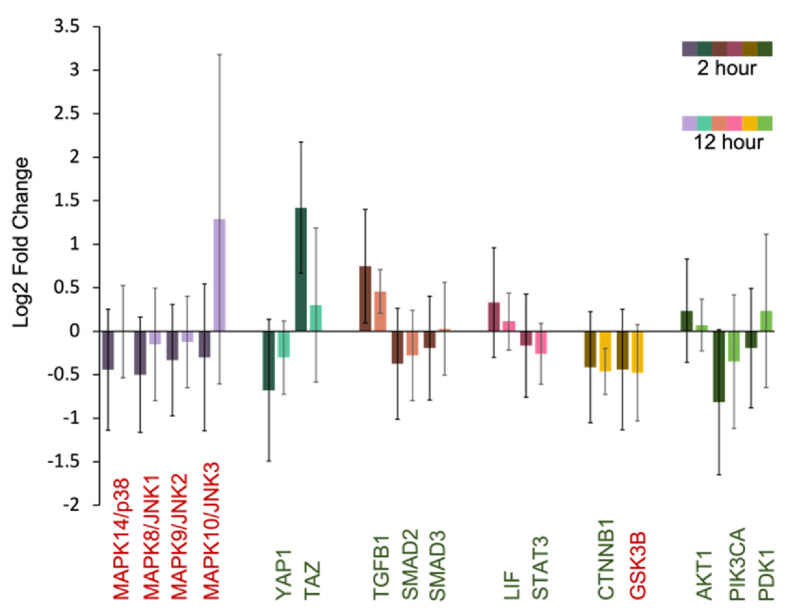
Differential expression of genes associated with pluripotency-relevant pathways. Genes that should be downregulated and upregulated are in red and green.

**Figure 5.**
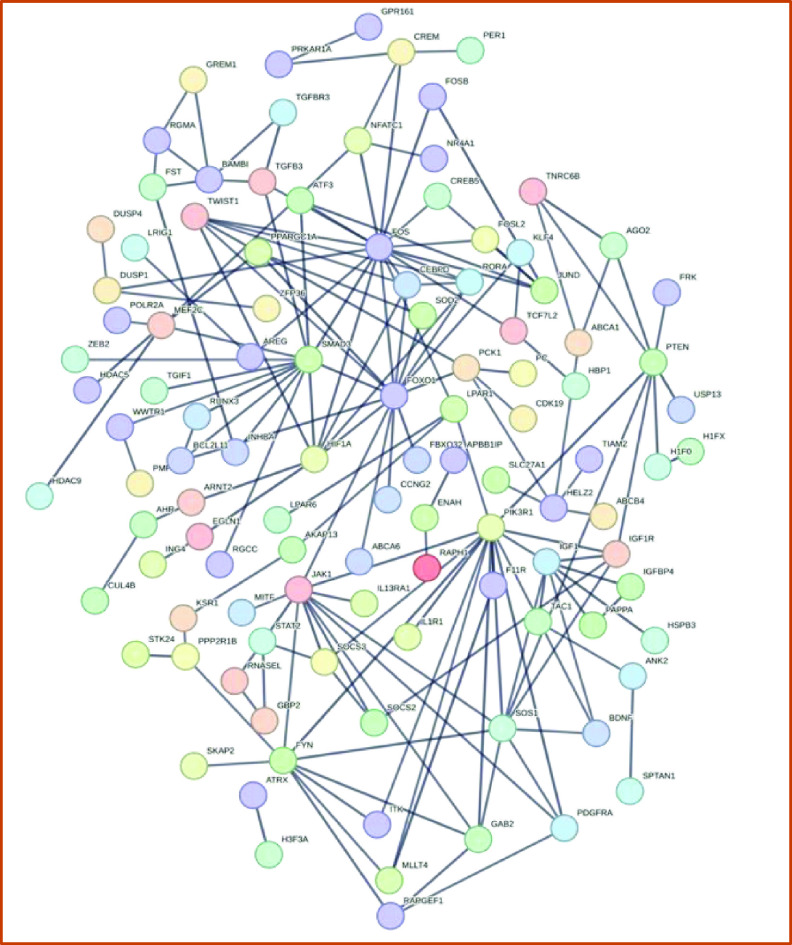
Significantly upregulated gene network diagram at 12 h, depicting the pivotal role of the pluripotency regulating genes FOS, FOXO1, and PIK3.

**Chart 1.**
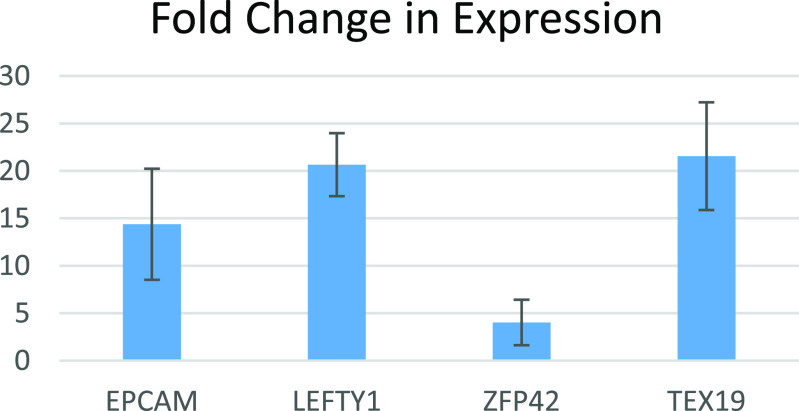
Fold-change in the expression of other pluripotency-related genes in TIB vs manually seeded fibroblasts.

Figure 4 shows the differential expression of genes associated with pluripotency-relevant pathways at 2 h and 12 h post-printing. Genes that have been shown to be downregulated and upregulated are in red and green respectively [14–16]. Figure 5 shows the gene network of significantly upregulated genes of the TIB cells 12 h post-printing and chart 1 shows other genes that were upregulated and are related to pluripotency.

## 4. Discussion

Preliminary results indicate that all cells expressed the three pluripotency markers oct-4, nanog, and sox-2. After applying a cardiomyocyte differentiation protocol, the cells stained positively for troponin-3. The cells also elongated and became more cardiomyocyte-like in their morphology. We analyzed bulk RNA seq data and our preliminary results show upregulation of some genes that have been implicated as stem cell markers: EPCAM, LEFTY1, ZFP42, and TEX19. In addition, differential expression of genes associated with pluripotency-relevant pathways shows some pathways are off like the MAPK/p38, MAPK/JNK1-3 which is expected for a pluripotent state. We also have data supporting the activation of the hippo pathway with TAZ highly upregulated and YAP staining the cell body. In addition, GSK3B is off and TGFB1, LIF/PIK3, and AKT1 are on as expected for pluripotency. Examining the gene network of upregulated genes, one can clearly distinguish the pivotal role of FOS, FOXO1, and PIK3 all related to pluripotency.

To our knowledge, this is the first reporting of this cell state, which we call strain-induced temporary auto-initiated reprogramming (SITAR). In our observations, this reprogramming is temporary, and with time, the expression levels of the three markers diminish. This can be appreciated by comparing the staining for Sox-2 or Nanog in the 3–4-day-old aggregates compared to the staining for these factors in freshly printed cells that were plated. However, after one week in pluripotent stem cell qualified culture, we could still observe some staining. Still, it appears only in the lower parts of the cell bodies or perhaps bound to the extracellular matrix gels, onto which the cells are plated. The temporary nature of the SITAR state would also explain the absence of reports in the bioprinting literature, even with bioprinting having become a widely used tool in many laboratories. Most groups will add specific media after bioprinting, which has been developed to maintain differentiated cells. We hypothesize that these media hasten re-differentiation from the SITAR state into the original cell type.

In order to begin to understand the origin of this discovery, we performed many basic experiments. It has been speculated that the heat supplied to the cells when using a thermal inkjet printer may be causing SITAR. This thermal shock was ruled out by manually jetting the cells through the orifices in the printhead, with no external power supplied and still observing the three pluripotent markers being expressed. However, when manually forcing the cell suspension through the orifices while immersing the printhead in culture media, SITAR is not observed. Thus further ruled out that shear experienced by cells as they pass through the 80 *µ*m orifices can account for the phenomenon. Therefore, our current understanding is that the formation of small droplets surrounding the cells is the triggering event that leads to the de-differentiation. The drop size in our printing setup is approximately 80 picoliters, and drops have very long and thin tails as they emerge from the orifice plate [17, 18]. It is plausible, that within these drops, the cell membranes get stretched and compressed within fraction of a second and that this sudden variation of strain is translated into the nucleus, where the expression of the more primitive or original genes is initiated. At this point, however, we do not have proof of this, and we are just reporting the state of the cells after 24 h to a few days post-TIB.

It should also be noted, that all the cells in the TIB samples are expressing OCT-4, most are expressing Sox-2, and somewhat fewer cells stain for Nanog. While more traditional methods of generating pluripotent stem cells are highly manual, with few cells needing to be hand-picked for culture, the TIB method is automated with no manual intervention needed, which may be an advantage for certain applications where fully automated systems are called for, in to large volume manufacturing for example.

Due to the transitory nature of the SITAR state, we have not observed stem cell-like characteristics being passed onto daughter cells after cell division. We do not know at this point if cultures could be propagated or not. TIB cells can be cryopreserved immediately after printing by adding 10% dimethyl sulfoxide (DMSO) to the media in the vials into which the cells are printed and slow cooling at 1 C per minute. In cryopreservation and thawing experiments, removing the cryoprotectant following standard protocols, the cells were still positive for the three markers tested.

SITAR cells are not truly pluripotent as far as we know at this point. For instance, even with the expression of markers indicating pluripotency, we have never seen the formation of teratomas in animals in our ten years of implanting TIB cells. However, recent preliminary experiments with the implantation of SITAR cell aggregates may show immature teratomas after 4 weeks of implantation; findings that will need to be repeated for longer periods of implantation. This, together with the seeming absence of colony formation and passing traits on to daughter cells, indicates to us that the state of SITAR cells is fleeting and non-stable, but may be prolonged by allowing aggregation of cells after bioprinting.

In summary, this temporary nature seems to open an opportunity for intervention, as the differentiation into a cardiac lineage seems to suggest. Developing and applying new differentiation protocols to SITAR cells may open opportunities in cell therapy that may not be not achieved otherwise.

## 5. Conclusions

We discovered that adult primary fibroblasts, when stretched inside 80 pl drops of fluid will at least temporarily adopt a more primitive or dedifferentiated state, reminiscent of pluripotency. The pluripotent-like cells can be differentiated into a cardiac lineage, amongst others. This phenomenon, which we call SITAR, is deemed to occur in all cells that experience the strain, and further exploring the SITAR state may lead to new insides about harnessing the inherent potential and regenerative medicine.

## Data Availability

The data that support the findings of this study are openly available at the following URL/DOI: https://minersutep-my.sharepoint.com/:f:/g/personal/tboland_utep_edu/EtIrJrZXQ2BOpiuk74MW3k4BISddXlhwQsmA/DedsShAxCg?e=UrpdQ4.
